# Supplementary material for: Homogeneous Chronic Subdural Hematoma with Diverse Recurrent Possibilities
Source: Diagnostics (Basel). 2022 Nov 4;12(11):2695. doi: 10.3390/diagnostics12112695 (PMC9689778; doi:10.3390/diagnostics12112695)
Supplement: Supplementary file 1 [file diagnostics-12-02695-s001.zip › diagnostics-1890256-supplementary.pdf]

Supplemental Table S1. The characteristic among 4 types of CSDHS.

	Trabecular	Homogenous	Laminar	Separate	P-value
Number	9	15	8	10	
Age	76.00 (13.50)	75.00 (12.00)	74.50 (8.50)	74.50 (13.75)	0.990
Gender					0.958
Male	7 (77.78%)	12 (80.00%)	6 (75.00%)	7 (70.00%)	
Female	2 (22.22%)	3 (20.00%)	2 (25.00%)	3 (30.00%)	
Follow up duration	51.00 (21.50)	52.00 (19.00)	47.50 (17.00)	46.00 (17.50)	0.794

Data are presented as median (IQR) or n (%).

Supplemental Table S2. The characteristic among 5 types of CSDHS.

	Homogeneous LD	Trabecular	Homogeneous HD	Laminar	Separate	P-value
Number	7	9	8	8	10	
Age	75.00 (18.00)	76.00 (13.50)	74.50 (11.25)	74.50 (8.50)	74.50 (13.75)	0.984
Gender						0.952
Male	5 (71.43%)	7 (77.78%)	7 (87.50%)	6 (75.00%)	7 (70.00%)	
Female	2 (28.57%)	2 (22.22%)	1 (12.50%)	2 (25.00%)	3 (30.00%)	
Follow up duration	53.00 (29.00)	51.00 (21.50)	51.00 (20.50)	47.50 (17.00)	46.00 (17.50)	0.904

Data are presented as median (IQR) or n (%).
